# Visible Light as an Antimicrobial Strategy for Inactivation of *Pseudomonas fluorescens* and *Staphylococcus epidermidis* Biofilms

**DOI:** 10.3390/antibiotics9040171

**Published:** 2020-04-10

**Authors:** Valeria Angarano, Cindy Smet, Simen Akkermans, Charlotte Watt, Andre Chieffi, Jan F.M. Van Impe

**Affiliations:** 1 BioTeC+, Chemical and Biochemical Process Technology and Control, Department of Chemical Engineering, KU Leuven, 9000 Gent, Belgium; valeria.angarano@kuleuven.be (V.A.); cindy.smet@kuleuven.be (C.S.); simen.akkermans@kuleuven.be (S.A.); charlotteg0306@gmail.com (C.W.); 2 Procter & Gamble, Newcastle Innovation Center, Newcastle NE12 9TS, UK; chieffi.a@pg.com

**Keywords:** Biofilm, photodynamic inactivation, visible light, light-emitting diode, polystyrene surface, inactivation kinetics, disinfection, antimicrobial inactivation, *Pseudomonas fluorescens*, *Staphylococcus epidermidis*

## Abstract

The increase of antimicrobial resistance is challenging the scientific community to find solutions to eradicate bacteria, specifically biofilms. Light-Emitting Diodes (LED) represent an alternative way to tackle this problem in the presence of endogenous or exogenous photosensitizers. This work adds to a growing body of research on photodynamic inactivation using visible light against biofilms. Violet (400 nm), blue (420 nm), green (570 nm), yellow (584 nm) and red (698 nm) LEDs were used against *Pseudomonas fluorescens* and *Staphylococcus epidermidis*. Biofilms, grown on a polystyrene surface, were irradiated for 4 h. Different irradiance levels were investigated (2.5%, 25%, 50% and 100% of the maximum irradiance). Surviving cells were quantified and the inactivation kinetic parameters were estimated. Violet light could successfully inactivate *P. fluorescens* and *S. epidermidis* (up to 6.80 and 3.69 log_10_ reduction, respectively), while blue light was effective only against *P. fluorescens* (100% of maximum irradiance). Green, yellow and red irradiation neither increased nor reduced the biofilm cell density. This is the first research to test five different wavelengths (each with three intensities) in the visible spectrum against Gram-positive and Gram-negative biofilms. It provides a detailed study of the potential of visible light against biofilms of a different Gram-nature.

## 1. Introduction

Biofilms are bacterial communities, mono- or multi-species, embedded in a self-produced matrix mainly composed of proteins, polysaccharides, extracellular DNA and lipids, which constitute the extracellular polymeric substance (EPS) [1,2,3,4,5,6]. The EPS allows bacteria to adhere to many surfaces and it protects the community from hazardous compounds. This crucial layer of protection decreases the bacterial susceptibility to disinfectants and antibiotics [7,8] and promotes cell communication via quorum sensing [9]. Biofilms living on surfaces have evolved to be more resistant to antibiotics, compared to their planktonic form [10,11]. They are identified as the major source of microbial contamination within the food industries, households and clinical environment [2]. Moreover, the development of antibiotic-resistant bacteria is raising a public health concern, and, at the same time, this underlines the urge for the scientific community to develop alternative antibacterial methods and strategies to fight infections [12]. Biofilms of different strains have been found in factories [13], meat, dairy, poultry industries [14], households [15], food processing industries [13] and hospitals [16]. They can resist harsh and hostile conditions, such as heat, low temperature, desiccation and shear forces, used as eradication methodologies [14,17]. Moreover, they cause issues ranging from process inefficiencies, such as corrosions or pipeline blockage [18], to infections due to their resistance to antibiotics [11].

Alternative, innovative and unconventional approaches are proposed to be used against microbial biofilms. These approaches can be based on the use of, e.g., plasma-based technologies [19], coatings embedding nanoparticles [20], ultrasound treatments [21], enzymatic disruption [22] or light-based technologies [23]. Within the light-technology framework, Light-Emitting Diodes (LEDs) are desirable for industries in terms of costs, safety, convenience and maintenance compared to other light sources, which have been used in different inactivation studies, e.g., lamps [24,25,26], lasers [27] or pulsed lights [28]. Moreover, they are characterized by narrow emission spectra allowing the selection of a specific range of wavelengths for irradiation. The use of visible light might represent an alternative way to effectively tackle biofilm problems, where antibiotics fail due to antimicrobial resistance. The photoeradication process is based on the absorption of photons by endogenous photosensitizers (PSs), when the microorganisms (fungi and bacteria) produce them, or exogenous PSs, when they are externally added before the irradiation process. After light excitation, the triplet state of these molecules results in the formation of reactive oxygen species [29,30,31]. The reactive oxygen species then cause damage to lipids, proteins, DNA, enzymes, RNA and other macromolecules and are therefore harmful to the microorganisms [32]. Some authors tried to quantify the endogenous PSs inside the bacteria [33,34,35,36], but the exact correlation between the light treatment efficiency and the number and type (structure, absorption and emission spectrum) of PSs within bacteria is still under investigation [37,38,39,40,41,42]. Based on some studies, the endogenous PSs absorbing light are ubiquinones, porphyrin-containing cytochromes or flavin-containing enzymes (having absorption spectra within 230–500 nm, 400–420 nm and 380–450 nm, respectively). More specifically, the bacterial porphyrines extracted and found in the literature are protoporphyin IX, zinc protoporphyrin IX, coproporphyrin III and uroporphyrin III, which can coexist within the same strain [37,38,41]. Different types imply distinct chemical structures, spectra and quantum yield of reactive oxygen species production.

The sole use of light as an antimicrobial technology is particularly advantageous because it exploits the endogenous PSs that are naturally present inside the cells. It omits the use of additional chemicals and antibiotics, categorizing the technology as environmentally friendly [37]. The technology has the potential to stop the dispersion of antibiotics within the soil, caused by animal manure, for instance. It could reduce the quantities absorbed by humans and animals due to their presence within the food chain [12]. Moreover, the disuse of chemicals and antibiotics encourages to reduce both the use of resources and the production of chemical waste, having an economic impact. The visible spectrum is stated as harmless to humans, unlike UVB (280–315 nm) which directly damages DNA of both microbial and human cells [23], and it makes the technology suitable for household, industrial and hospital applications. Finally, some studies demonstrated that the light treatment does not cause the development of antimicrobial resistance thanks to the multi-target mechanism causing cell death [25,37,43,44]. The number of photodynamic inactivation studies has greatly increased in recent years. These studies have mainly examined planktonic cells [38,39,40,41,43,45,46,47,48,49,50,51,52], while few studies have taken bacterial biofilms into consideration [16,30,32,50,51,52,53,54,55]. Since biofilms pose a major global healthcare problem, the impact of photodynamic inactivation on them is both crucial and innovative. Previous research has found a bactericidal/inhibitory effect of light within the visible range on different species (*Pseudomonas*, *Staphylococcus*, *Escherichia*, *Listeria*, etc.) [27,30,32,46,48,49,50,51,56]. However, these studies are mainly focused on blue and violet light, even if there is previous evidence in the literature that green, yellow and red light had an impact on several bacterial species and fungi either promoting growth [49,57,58] or decreasing the bacterial population [24,30,46,48,49].

Since biofilms can directly be treated with a light source, regardless of the shape and the material composing the surface, the light treatment becomes simply applicable and advisable as new technology [51,59]. Indeed, this study adds to a growing body of research on photodynamic inactivation using visible light against biofilms. Thus, the impact of violet (400 nm), blue (420 nm), green (570 nm), yellow (584 nm) and red (698 nm) light with different irradiance levels (25%, 75% and 100% of maximum irradiance (I_max_) for all wavelengths and 2.5% for violet as well) was examined.

*Pseudomonas fluorescens* and *Staphylococcus epidermidis* were selected as model organisms. Their choice was based on the interest to test two different species in terms of Gram-nature (a Gram-positive and a Gram-negative), but also on their relevance in several settings: hospitals, industries and households. *Pseudomonas* spp., indeed, are mostly studied in biofilm processes. *P. fluorescens*’ relevance specifically stems from being the root of significant contamination problems within the food industry (dairy and meat) to agricultural microbiology. In 2010 for instance, a lot of food wastage in both Italy and Germany occurred because of *P. fluorescens*, a blue-pigment producer, which caused blue discoloration of mozzarella [60]. *S. epidermidis* is an opportunistic pathogen that was chosen for its large presence on skin and as well as its relevance within nosocomial biofilm-related infection [61]. Biofilms grown on a flat polystyrene surface were used to investigate the photodynamic effect. The development of biofilms on a flat surface rather than in a 96-well plate promotes the direct photon–biofilm interaction, avoiding shadow areas or regions. The choice of polystyrene (PS) was made based on the lack of specific surface chemistry to make the study general for different applications, processes and systems under control. Moreover, PS is a material of a large relevance in hospital settings, the food industry and households. The inactivation kinetics were studied by using the model of Geeraerd et al. to describe the results, estimate the inactivation parameters and make a comparison of the most effective treatments [62]. The log_10_ reductions were evaluated after each treatment and the influence of the light irradiance (inactivation by light), dosage (dose for 1-log_10_ reduction) and time exposure were studied to achieve an improved understanding of the antimicrobial light-based approach. The experiments were performed on general and selective media as well, to check if the light causes sublethal injury within the biofilms. This is the first research to test five different wavelengths distributed in the visible light spectrum, each with three different irradiance levels (four for violet light), on both Gram-positive and Gram-negative biofilms. As such, this research provides a broad study of the potential of visible light as an antibiofilm treatment.

## 2. Results

Biofilms of *P. fluorescens* and *S. epidermidis*, grown on polystyrene, were exposed to colored LED arrays (violet, blue, green, yellow and red). Different irradiances were tested (2.5%, 25%, 75% and 100% of I_max_). The Geeraerd et al. model was fitted to experimental data [62]. Specifically, for *P. fluorescens* (for all the light colors and conditions) and *S. epidermidis* for the violet light, the Geeraerd et al. model without shoulder was used, while for *S. epidermidis* for the blue, green, yellow and red light, the model without shoulder and tail, which reduced to a log-linear regression model, was employed. The model allowed for the estimation of the inactivation parameters, i.e., initial population *N*_0_, residual population *N_res_* (if applicable depending on the model) and inactivation rate *k_max_*. Sublethal injury kinetics, expressing the sub-lethally injured cell percentages within the biofilm due to the treatment, were calculated using Equation (6) for both bacterial biofilms.

### 2.1. Light Characterization

The LED arrays were optically characterized. The emission spectra were measured, and they were found to have an emission peak (λ_max_) at 400, 420, 570, 584 and 698 nm for violet, blue, green, yellow and red, respectively. The values of full width at half maximum, Δλ, ranged from 20 to 95 nm (details are reported in Table 1). The power densities emitted by the LED when the percentage of I_max_ was set at 2.5%, 25%, 75% and 100% are listed in Table 1 and ranged from 0.043 to 29.2 mW cm^−2^. After 4 h of light exposure, the total dose received by the biofilms was calculated using Equation (1). It ranged from 10.1 to 420.5 J cm^−2^ for violet, from 2.5 to 6.9 J cm^−2^ for blue, from 1.6 to 3.7 J cm^−2^ for green, from 0.6 to 2.0 J cm^−2^ for yellow and from 1.7 to 5.0 J cm^−2^ for red LED array. The violet LED array emitted the highest irradiance and 2.5% I_max_ was adopted to have irradiances comparable to the remaining LED arrays. The detailed irradiances and doses for each condition are also reported in Table 1.

### 2.2. Differences in Microorganisms’ Biofilms

**Figure 1:**
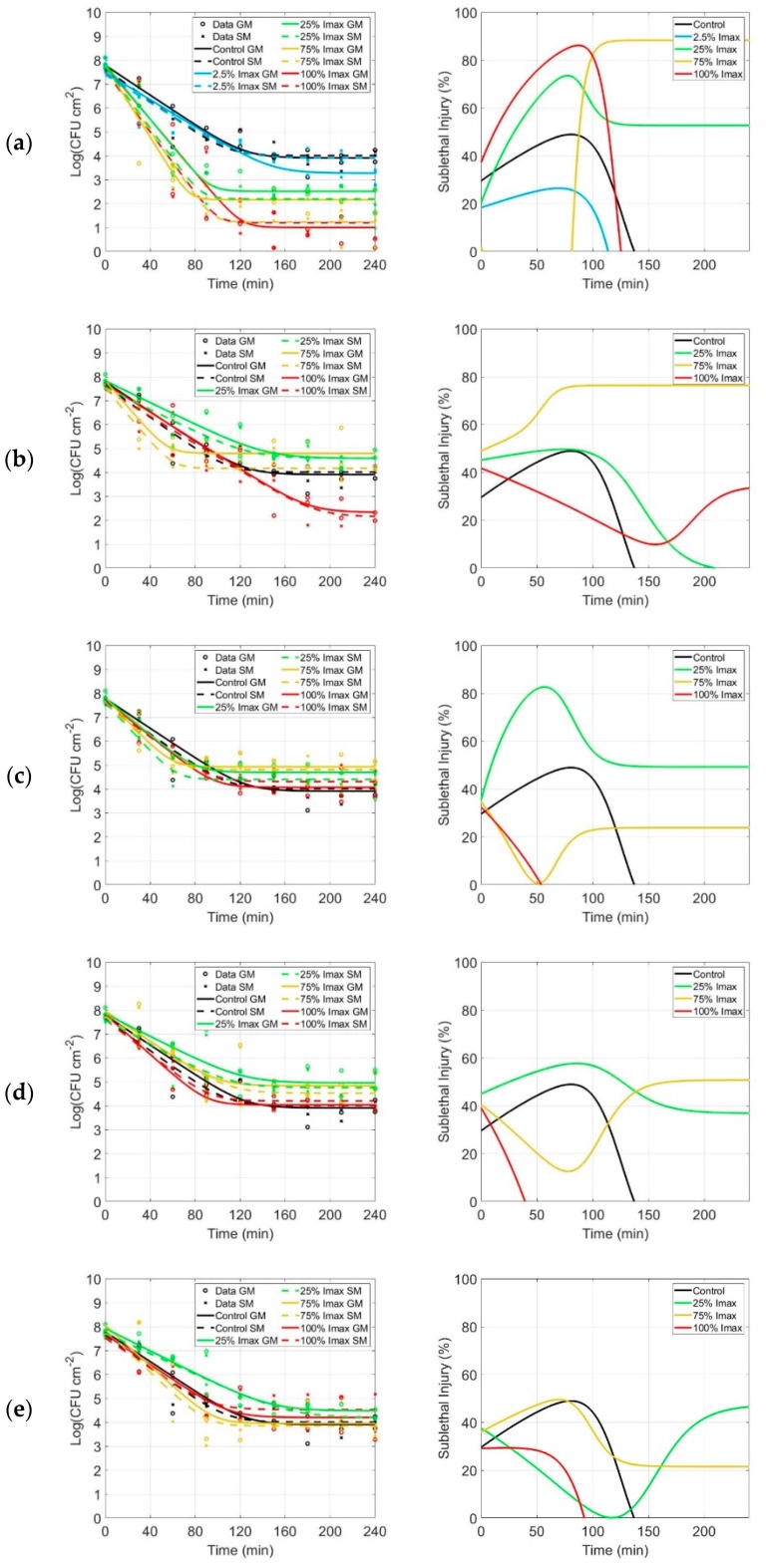
Inactivation and sublethal injury kinetics of *P. fluorescens*. Inactivation kinetics (first column) and sublethal injury (SI) kinetics (second column) for *P. fluorescens* biofilms following irradiation with (**a**) violet, (**b**) blue, (**c**) green, (**d**) yellow and (**e**) red LED arrays. Inactivation kinetics: the black symbols (o for general medium,GM, and x for selective medium, SM) and lines (solid line for GM, dashed line for SM) represent the control (no light exposure). Likewise, the colored symbols and lines represent the fitting after 2.5% (only for violet LED array, light blue), 25% (yellow), 75% (green) and 100% of I_max_ (red). Sublethal injury kinetics: the colored lines represent the SI after 2.5% (only for violet LED array, light blue), 25% (yellow), 75% (green) and 100% of I_max_ (red).

**Figure 2:**
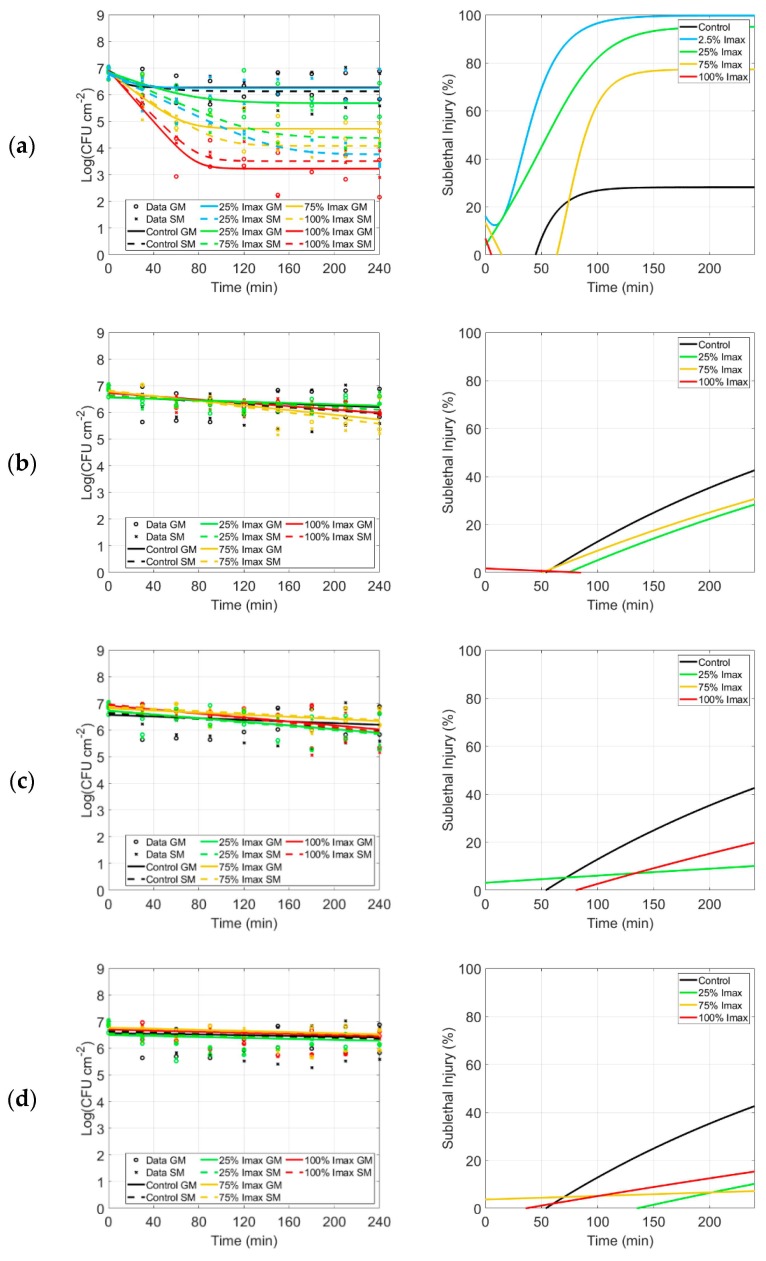

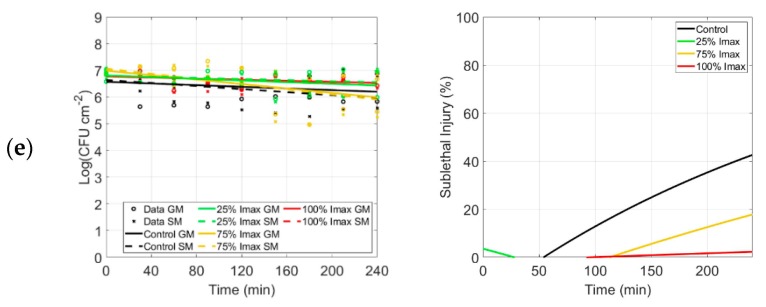
Inactivation and sublethal injury kinetics of *S. epidermidis*. Inactivation kinetics (first column) and sublethal injury (SI) kinetics (second column) for *S. epidermidis* biofilms following irradiation with (**a**) violet, (**b**) blue, (**c**) green, (**d**) yellow and (**e**) red LED arrays. Inactivation kinetics: the black symbols (o for GM, x for SM) and lines (solid line for GM, dashed line for SM) represent the control (no light exposure). Likewise, the colored symbols and lines represent the fitting after 2.5% (only for violet LED array, light blue), 25% (yellow), 75% (green) and 100% of I_max_ (red). Sublethal injury kinetics: the colored lines represent the SI after 2.5% (only for violet LED array, light blue), 25% (yellow), 75% (green) and 100% of I_max_ (red).

Two model biofilms were developed from two different bacteria: *P. fluorescens*, which are Gram-negative bacteria, and *S. epidermidis*, which are Gram-positive ones. Although the mono-species biofilms were grown using the same methodology, after 24 h of growth, the resulting biofilms were slightly different in (i) Colony Forming Units (CFU) per area and (ii) resistance to desiccation, perhaps due to the difference in Gram classification. In fact, (i) 24 h-grown *P. fluorescens* biofilms reached a population of approximately 10^8^ CFU cm^−2^, while *S. epidermidis* biofilms had a population of 10^7^ CFU cm^−2^ (these population densities were then exposed to light treatment). Moreover, (ii) *P. fluorescens* biofilms were more sensitive to evaporation of water that caused some cells to die because of drying (black line and dots in Figure 1). On the contrary, *S. epidermidis* biofilms showed no reductions due to drying (black line and dots in Figure 2). The population of the *S. epidermidis* biofilm also remained constant after 4 h.

### 2.3. P. Fluorescens

#### 2.3.1. Inactivation Kinetics, Parameters and log_10_ Reductions

The inactivation kinetics obtained by irradiating *P. fluorescens* biofilms utilizing violet (Figure 1a), blue (Figure 1b), green (Figure 1c), yellow (Figure 1d) and red (Figure 1e) light are presented in Figure 1. Different irradiation conditions were tested for each color (2.5% for violet only, 25%, 75% and 100% of I_max_). *N*_0_, *N_res_* and *k_max_* were estimated and their respective standard errors (SE) and the root mean squared error (RMSE) values were calculated. These values were summarized in Table 2, along with the statistical analysis. The overall log_10_ reductions (*N*_0_ − *N_res_*) were also calculated and are listed in the same table.

The inactivation kinetics are characterized by a log-linear inactivation phase, from time 0 to approximately 90–100 min, followed by a tailing phase (Figure 1). The log-linear inactivation is present in the control as well, due to a desiccation process occurring to the biofilms within the first 100 min.

Regardless of the treatment, the rate of inactivation *k_max_* was in general very similar to the control (no significant differences), as can be noticed from the *k_max_* values displayed in Table 2. This means that the inactivation rate was not influenced by the light exposure, while the residual population *N_res_* was strongly influenced in some conditions.

Different percentages of I_max_ (2.5% for violet only, 25%, 75% and 100% of I_max_) per LED color resulted in higher or equivalent log_10_ reductions compared to the control (no light exposure). The log_10_ reductions indicated that the highest reductions were obtained with violet light (in all conditions) and blue light when I_max_ equaled 100%. Indeed, the most effective inactivation was observed when the violet array was tested on biofilms. This resulted in a log_10_ reduction ≤ 6.80 CFU cm^−2^, while the reduction of the control was only 3.87 CFU cm^−2^ (due to natural desiccation). A log_10_ reduction ≤ 5.37 CFU cm^−2^ was induced for biofilms exposed to blue LED irradiation. Furthermore, experiments were carried out with green, yellow and red irradiation. These revealed no additional reduction compared to the control.

#### 2.3.2. Sublethal Injury

The model was also fitted to the data resulting from the selective plating media. The inactivation kinetics (dashed lines) and the parameters, related to selective media and estimated from the Geeraerd et al. [62] model, are displayed in Figure 1 and Table 2. The sublethal injury of *P. fluorescens* was determined by plating the bacterial suspension on general (GM) and selective media (SM) to quantify the sublethal cell damage after irradiation. Preliminary experiments were carried out to check that SM did not affect the growth of the cells of the biofilms after mechanical detachment of the biofilm using a scraper. The SI curve related to the control experiments (no light exposure) showed a peak at about 50%. This corresponds to the phase-change between the log-linear phase and the tail in the inactivation curve (column 1, Figure 1). As violet exposure is tested, SI curves have a higher peak corresponding to 25% and 100% I_max_ and a lower one corresponding to 2.5% I_max_ in the same time frame. 75% I_max_ is an exception, showing a plateau corresponding to the tail of the inactivation curve. Generally, the other SI curves have a similar trend compared to the control, except for 75% I_max_ with blue and green exposure.

### 2.4. S. Epidermidis

#### 2.4.1. Inactivation Kinetics, Parameters and log_10_ Reduction

For *S. epidermidis* biofilms, similar experiments were carried out. Figure 2 displays the inactivation kinetics obtained using violet (Figure 2a), blue (Figure 2b), green (Figure 2c), yellow (Figure 2d) and red (Figure 2e) LED arrays and the models fitting the data. The estimated parameters *N*_0_, *N_res_* (if applicable depending on the model) and *k_max_*, the respective SE, RMSE values and the log_10_ reduction are summarized in Table 3, for both models. As in the previous *P. fluorescens* experiment, different irradiation conditions (2.5% for violet only, 25%, 75% and 100% of I_max_) were tested for each color.

For biofilms exposed to violet light, the inactivation kinetics were characterized by a log-linear inactivation phase, lasting approximately 80 to 90 min, after which a tailing phase followed (Figure 2). The control did not undergo a desiccation process as seen with *P. fluorescens*. The initial population was approximately 6.84 CFU cm^−2^ and the residual population was almost the same (6.27 CFU cm^−2^). The standard errors of the inactivation rate, *k_max_*, demonstrate that this parameter could not be estimated accurately. Based on the parameters’ estimation, the log_10_ reduction was determined and had a much better accuracy. When blue, green, yellow and red LED arrays were used (in all conditions), the cells within the biofilm after 4 h of treatment were almost the same compared to the control, and the model without shoulder and without tail (log-linear regression) was used to fit the data and represent the kinetics. The rates of inactivation, *k_max_*, did not show any increase for these LED array exposures (Table 3).

A log_10_ reduction of approximately 3.69 CFU cm^−2^ was induced by violet exposure (harshest condition) compared to approximately 0.57 CFU cm^−2^ of the control. Regarding biofilms exposed to other colors (blue, green, yellow and red), no substantial reduction was observed in terms of biofilm populations (Figure 2). The estimated reductions for each condition are reported in detail for both general and selective media in Table 3.

#### 2.4.2. Sublethal Injury

The different evolution in SI for controls of *S. epidermidis* for violet light (Figure 2a) and blue, yellow, green and red light (Figure 2b–e) were caused by the use of two different (simplified) versions of the Geeraerd et al. model (as explained in Section 4.7). With regards to SI kinetics related to violet light, the maximum SI value decreases (100%, 95% and 75% related to the plateau values) as the irradiance increases from 2.5% to 75% I_max_ (Figure 2). This means that the stronger the irradiance, the stronger the killing effect. Indeed, in the harshest condition, the majority of the cells are dead rather than damaged. No evidence of this trend is present when I_max_ equals 100%. Instead, SI curves lay underneath the control for blue, green, yellow and red exposures. Negligible SI values were observed for those treatments.

### 2.5. Inactivation by Light and Dose for 1-log_10_ Reduction

**Figure 3:**
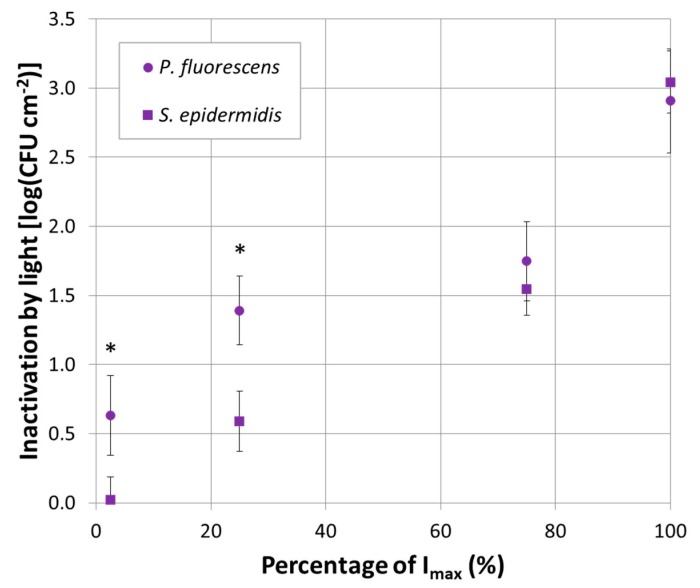
Violet LED treatments. Inactivation by light of *P. fluorescens* (round symbols) and *S. epidermidis* (squared symbols) under violet exposure with 2.5%, 25%, 75% and 100% of I_max_. The * symbol indicates significant differences (*p* < 0.05).

To understand the mere effect of the irradiation on biofilms, without considering the effect of the desiccation that results in a reduction of the population of *P. fluorescens* (Section 3.2), the inactivation by light itself was calculated (*N_res_ control – N_res_ light*). This data analysis concerns the fits obtained with violet-exposed biofilms, for which the residual population was estimated through the Geeraerd et al. model [62]. In Figure 3, the population of bacteria killed employing violet light exposure (*N_res_ control – N_res_ light*) was reported as a function of the percentage of I_max_. Figure 3 demonstrates that light treatments with 100% and 75% I_max_ were found to cause a reduction of approximately 3.0 log_10_ and 1.5 log_10_ CFU cm^−2^ in the population of both *P. fluorescens* and *S. epidermidis* biofilms. Whereas 25% and 2.5% I_max_ caused a reduction of approximately 1.39 and 0.63 log_10_ CFU cm^−2^ for *P. fluorescens* respectively, and for *S. epidermidis*, 0.59 and 0.02 log_10_ CFU cm^−2^, respectively.

**Figure 4:**
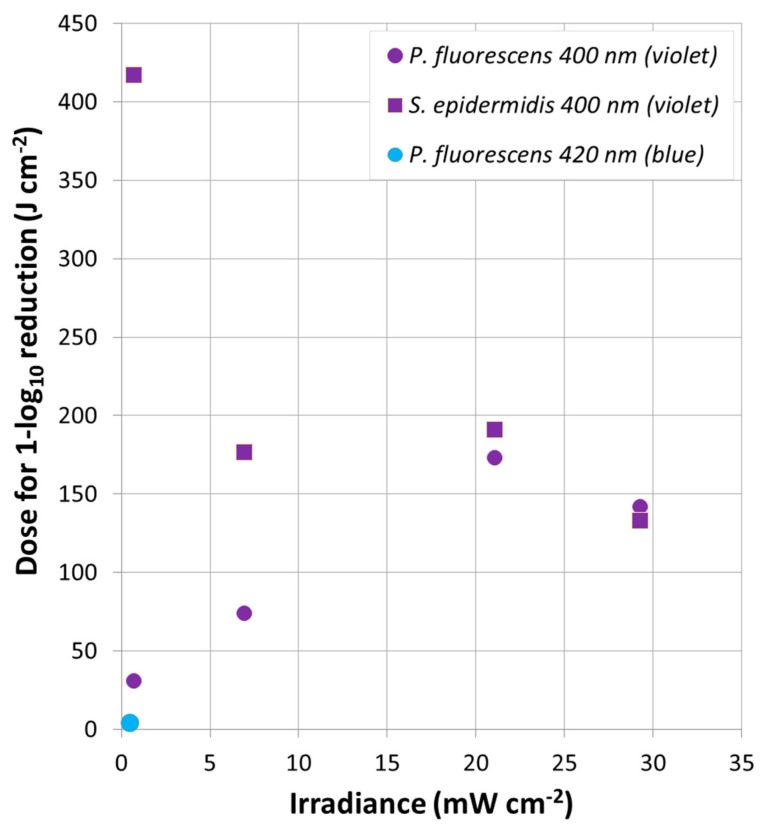
Dose for 1-log reduction. Dose for 1-log_10_ reduction as a function of the irradiance. *P. fluorescens* (round symbols) and *S. epidermidis* (squared symbols) are represented in violet color for 400 nm. The treatment with 100% of I_max_ using 420 nm (blue light) is represented in blue (only for *P. fluorescens*).

Moreover, the dose for 1-log_10_ reduction was calculated following Equation (6). It represents the required dose to produce 1-log_10_ inactivation in the population of the specific bacterial strain. It was estimated for each violet-investigated condition. It is displayed for both *P. fluorescens* and *S. epidermidis* as a function of the irradiance (Figure 4). The most effective treatments (violet treatments and 100% I_max_ with blue light on *P. fluorescens*) were considered in this analysis.

Figure 4 reveals that the dose required for 1-log_10_ reduction was ≥ 31 J cm^−2^ for *P. fluorescens* and ≥ 133 J cm^−2^ for *S. epidermidis*, with 400 nm peaking light (violet). However, *P. fluorescens* required only 4 J cm^−2^ when blue light was used. Figure 4 also displays the specific bacterial trends. Regarding *P. fluorescens* inactivation (Figure 4, round legend symbols), a little increase in dose for 1-log_10_ reduction was required as irradiance increased. However, an opposite trend was evident in *S. epidermidis* data (Figure 4, squared legend symbols), where a higher irradiance resulted in a decrease of the dose for 1-log_10_ reduction.

## 3. Discussion

This study investigated the antibacterial effect of a high-tech optical set up, composed of an exchangeable LED array emitting in the visible range, on *P. fluorescens* and *S. epidermidis* biofilms. The impact of several LED arrays, characterized by different wavelengths and irradiances, was examined. In this research, biofilms were grown on a plastic (polystyrene) surface to facilitate the extension of this study to real contaminated surfaces within the food, healthcare, households and hospital settings [58]. Future studies should account for different (bio)materials and assess the photodynamic inactivation impact on non-flat surfaces as well.

### 3.1. P. fluorescens and S. epidermidis Inactivation by LEDs

The most evident outcome, resulting from the experiments carried out, was the inactivation of the Gram-negative *P. fluorescens* and Gram-positive *S. epidermidis* biofilms, utilizing violet LED light. In contrast, the performed experiments proved neither promotion nor reduction of biofilm inactivation when green, yellow and red light exposure was employed. Blue light had an exceptional impact depending on the bacterial biofilms which were tested: it had a killing effect on *P. fluorescens* (with 100% of I_max_) while it was harmless for *S. epidermidis*.

#### 3.1.1. Violet Light

A previous study, conducted with violet light (405 nm LED) on planktonic bacteria, found a reduction of 1.0 log_10_ within the population [52]. The bacteria undergoing the treatment were the food pathogen *Staphylococcus aureus*, while refrigerated (4 °C), for a treatment time of 7.5 h and a total dose of 486 J cm^−2^. The same authors attributed the antibacterial effect induced by the LED light to physical damage of the bacterial wall rather than to DNA destruction: DNA was found not to be damaged [52]. Ferrer-Espada et al. who focused their work on biofilms grown using a CDC reactor, found a reduction of 1.88, 2.78 and 3.18 log_10_ for *Acinetobacter baumannii*, *Escherichia coli* and *Pseudomonas aeruginosa* irradiated with 405 nm [32]. They exposed biofilms to doses of 162, 576 and 500 J cm^−2^ respectively, compared to 420 J cm^−2^ used in the current research. Inactivation was claimed by Halstead et al. as well, who investigated 400 nm light against 34 isolates and found violet light effective against *P. aeruginosa* and *S. aureus* [53]. Based on these findings and the literature, there is no doubt about the antibacterial activity of the violet light [32,40,41,43,49,51,54,56,63,64,65,66,67]. However, the evaluation of its effectiveness and a direct comparison of the results, obtained by different studies, are more complicated. The complexity is due to the usage of planktonic suspensions rather than biofilms, different protocols used to grow biofilms, different initial populations, models used for the inactivation, exposure times, supplied doses, irradiances and, finally, specific bacterial properties and features (PSs contents and types) under investigation. In this work, the possibility to change the irradiance emitted by the set up allowed for the investigation of several irradiances. It was found that the stronger the violet irradiance became, the lower the residual population, *N_res_*, was. This was valid for both examined bacteria and it is in complete agreement with the literature focused on the UV range, where powerful sources promoted complete eradication [23]. The findings of Section 2.5 also demonstrated that inactivation (light-killed population) of *P. fluorescens* biofilms was stronger than *S. epidermidis* biofilms when the total dose irradiating the samples was lower (2.5% and 25% I_max_, Figure 3 in Section 2.5). It can be conceivably hypothesized that the difference between *P. fluorescens* and *S. epidermidis* inactivation may be due to the presence of different endogenous PSs within each bacterial species having different absorption spectra [38,39,40,41,47]. Another reason may be the different quantity of PSs absorbing light per bacterial cell: a higher concentration leads to increased reactive oxygen species production and, therefore, a stronger killing effect under lower irradiation conditions. The level of endogenous PSs is indeed an intrinsic characteristic of the strain. Moreover, the probability of photoexcitation of a PS is driven by the direct interaction, photons–PS. Because the two species are characterized by a different cell wall as a result of the Gram-nature, the physiological and morphological structure of the bacteria played a role as well. This aspect is discussed in Section 3.3.

#### 3.1.2. Blue Light 

In some works, blue light (420–470 nm) has been tested as well, showing a strong effect against both Gram-positive (*Bacillus cereus*, *Listeria monocytogenes* and methicillin-resistant *S. aureus*) and Gram-negative pathogens (*P. aeruginosa*, *Salmonella* Typhimurium and *E. coli*) in their planktonic form [39,40,41,49,50,54,56,65,66,67,68]. However, Abana et al. claimed that the effect of 455 nm is not completely bactericidal on *E. coli* but depends on the microorganism (pathogenic or non-pathogenic) and growth phase. They demonstrated that the stationary phase was more resistant compared to the exponential phase [45]. Based on our findings, blue light only had an impact on *P. fluorescens* biofilms under the harshest condition (100% I_max_). No evidence of inactivation was observed on *S. epidermidis* biofilms. The inactivation obtained with the blue light on *P. fluorescens* biofilms correlates favorably with Amin et al. and further supports the idea that *P. fluorescens* likely contains porphyrins absorbing specific wavelengths of the blue emission spectrum (420 nm for instance) that might promote cell death [37]. Hyun and Lee observed that *P. fluorescens* compared to *L. monocytogenes* was found to be the most sensitive strain to blue light treatment [50]. However, these authors were using a blue light peaking at 460 nm and investigating the effect on bacteria inoculated and left dry on packaging materials (no biofilms). The light provoked disruption of the cell membrane, and release of intracellular component post-treatment [50].

#### 3.1.3. Green, Yellow and Red Light

The poor performances (unaltered population density) obtained with green, yellow and red light exposure in terms of inactivation were in contrast with some works that, instead, evidenced the influence of this visible range on bacteria. Some studies performed on planktonic form showed reduction or promotion on bacterial cells depending on the temperature at which the experiments were performed. Ghate et al. investigated the impact of 521 and 642 nm on four different pathogens at different temperatures (20, 15 and 10 °C) for a total exposure time of 7.5 h [49]. At 20 °C, they found an increase of the population densities (approximately 1.5 log_10_) when the four bacteria were exposed to one of these two wavelengths. At 15 and 10 °C, instead, a reduction was observed when bacteria were irradiated at 521 nm. Specifically, 1.7, 1.7, 1.0 and 0.9 log_10_ at 15 °C and 1.8, 1.7, 1.5 and 1.5 log_10_ 10 °C for *S.* Typhimurium, *S. aureus*, *E.coli* and *L. monocytogenes*, respectively. No variation within the population densities was found at 642 nm.

Other studies showed that the bacterial species under investigation played a crucial role on the inactivation. In fact, Kim et al. found a reduction up to 60% and 40% on *S. aureus* and *E. coli*, respectively [48]. On the contrary, they found an increase of the population of 2.5-fold after 8 h for *Pseudomonas gingivalis.* These experiments were performed with irradiation at 525 nm on planktonic cells. Yu and Lee, on one hand, found that green light promoted the antifungal activity of *Bacillus amyloliquefaciens* but, on the other hand, claimed that it reduced mycelial growth on *Colletotrichum acutatum* [57,58].

Then, there are other studies where a reduction is observed regardless of the two above-mentioned temperature and species. Ghate et al. studied the effect of 461 and 521 nm against the pathogens *E. coli*, *S.* Typhimurium and *L. monocytogenes* [46]. Treatments of 7.5 h were applied with a total dose ranging from 100 to 600 J cm^−2^ and led to the conclusion that 521 nm decreased the bacteria in their planktonic form. The efficiency of the green light treatment was lower than blue light but still, it caused a reduction of 1–2 log_10_ CFU mL^−1^ at both acidic and alkaline conditions. Kumar et al. also demonstrated at 520 nm a reduction of 1.2 log_10_ at 25 °C of *S. aureus* and 0.7 log_10_ at 25, 10 and 4 °C of *L. monocytogenes*. Some studies performed on fungi showed similar impacts [30].

Brito Aragão et al. focused, instead, on biofilms. They tested two different red peaking LEDs with an emission range of 620–660 nm and found that irradiation alone was causing no reduction within the population [55], while Yu and Lee found enhanced biofilms’ formation and motility on *B. amyloliquefaciens* [57]. Even though these wavelengths were chosen because they showed growth enhancing or bactericidal effects, none of these effects was observed against biofilms of *P. fluorescens* and *S. epidermidis* under different conditions. This research demonstrated the ineffectiveness of these wavelengths in terms of inactivation of two specific bacterial biofilms. Future research should be focused on the use of the examined ineffective wavelengths to promote antibacterial activity in synergy with exogenous PSs [27,44,69,70].

### 3.2. Sublethal Injury

Although the sublethal injury (SI) kinetics have completely different trends and percentages of injury, confirming the response to the treatments depends on the microorganism, a generalization on the light impact is possible. The SI curves for violet LEDs demonstrate that for both microorganisms, the percentages of SI increase when the irradiance increases. This can be easily explained: the number of photons reaching the biofilms is higher and consequently, so is the sublethal injury. However, the tendency of the SI is slightly different compared to what was found in other research. Ghate et al. determined that the SI only increased with the exposure time [46]. In contrast, in this study, it reaches a plateau or a peak. This is probably due to the difference in population growth forms: planktonic compared to biofilms.

### 3.3. Factors Influencing the Inactivation Kinetics

Several factors influenced the kinetics of the photoinactivation: (i) the Gram-nature and the intrinsic difference within the developed biofilms belonging to the two species, (ii) the endogenous PSs within microorganisms, (iii) the direct impact on enzymes and (iv) the production of specific molecules by microorganisms.

First of all, regarding the Gram-nature, an important result that emerged from this study is that Gram-negative *P. fluorescens* and Gram-positive *S. epidermidis* biofilms, responded with the same photoinactivation to a treatment with violet light at the two highest irradiances (100% and 75% of I_max_): for both species, populations were inactivated with 3 log_10_ and 1.5 log_10_ without any significant differences between the two microorganisms (Section 2.5). These findings are in contradiction with previous studies reported in the literature, where some authors confirmed a stronger inactivation effect on Gram-positive microorganisms, which have a different cell wall lacking both outer membrane and lipopolysaccharide layer but have a thicker peptidoglycan layer [51]. They are also in contrast with others claiming that the most sensible bacteria are the Gram-negative ones, even though they are characterized by a more complex cell wall [53]. Halstead et al. found that among 34 isolates, the most susceptible were the Gram-negative bacteria *A. baumannii* ACI_19606, for which a 93.5% reduction was observed after 15 min of light exposure [53]. The intrinsic differences between the two types of bacteria (Gram-nature) and the biofilm they formed is also an interesting aspect. A comparison between the curves of the controls for *P. fluorescens* and *S. epidermidis* showed how the biofilms, developed using the two strains, reacted differently under drying conditions. *P. fluorescens* underwent a process of desiccation that can justify the reduction in the biofilm population (no light-exposure), while the *S. epidermidis* biofilms did not show any reduction. Gram-negative bacteria seemed more sensitive to drying than Gram-positive, as previous authors confirmed [15]. The comparison between the kinetics of *P. fluorescens* and *S. epidermidis* requires further comments. Although the inactivation kinetics are alike in shape, they are slightly different regarding the time frame of the log-linear phase, which was shorter for *S. epidermidis* compared to *P. fluorescens*.

Then, based on the literature that focused on the investigation of the endogenous PSs responsible for the photoinactivation and considering the absorption spectra of the porphyrins and flavins, it is possible to claim that the wavelengths with the strongest antibacterial effect are the ones belonging to the main absorption peak: violet-blue range [38,39,40,41,47]. Although the other wavelengths of the visible spectra followed in correspondence with the secondary absorption peaks of the same chromophores, they were harmless [38,39,40,41,47].

Another factor that can be involved is the direct damage caused by photons to the enzymes succinate dehydrogenase (SDH) and lactate dehydrogenase (LDH). This process does not involve reactive oxygen species production [71]. The damage mechanism is due to the tail of the violet LED spectrum continuing in the UV-A spectral region, which is responsible for their destruction [71]. Finally, the last factor that may play a role in the detrimental effect of light on *P. fluorescens* biofilms is its effect on the production of pyocyanin or pyoverdine (siderophores), blue extracellular products that may contribute to light absorption [67,72].

### 3.4. Doses and Bacterial Trends

The analyses of the dose required for 1 log_10_ reduction highlighted the impact of violet or blue exposure on *P. fluorescens* and *S. epidermidis* biofilms as antibacterial treatment, compared to the other ineffective wavelengths (blue, green, yellow and red). Concerning *P. fluorescens*, even better inactivation was achieved with blue light rather than violet (Figure 4). A remarkable difference in the dose required for 1 log_10_ reduction between blue and violet light was observed. The blue dose required to reduce the bacteria population in the biofilm was found to be 8 times higher compared to the violet dose (Section 2.5). A lower dose means lower energy requirement per area and translates into lower energy consumption and lower costs, thus representing an advantage as new technology.

The examination of the bacterial trends (dose for 1 log_10_ reduction as a function of irradiance) demonstrated interesting features. It was found that the dose for 1 log_10_ reduction increases asymptotically as the irradiance increases for *P. fluorescens* biofilms, whereas it decreases asymptotically as the irradiance increases for *S. epidermidis* biofilms. Tomb et al. observed the opposite trend for *P. fluorescens* (required dose decreases as a function of irradiance), although *S. epidermidis’* trend is in line and practically the same as the one proposed by the same authors [67]. It was hypothesized that the opposite trend might be due to a saturation effect. The excess of irradiance does not enhance the number of excited endogenous PSs within *P. fluorescens* cells that might be previously promoted to an excited molecular state [73].

The presence of a tail in the kinetics, obtained using the Geeraerd et al. model, made clear that the inactivation with violet light after 180 min can no more and no longer increase the number of dead cells [62]. An increase in the exposure time after a certain cut off value is useless and does not promote any additional activation. This is an important aspect that is first different in planktonic form where cells are free to float [46], compared to the situation where the cells are trapped within the matrix (biofilm). Moreover, the exposure time in this work is an average between the long treatments, such as the 4 days of exposure tested by Hyun et al. against *P. fluorescens* and *L. monocytogenes* [50] and the short exposures, like Rupel et al. who used treatment times of a few minutes. The latter used a powerful laser source at 455 nm, assuring an effect on a short time but using a light source characterized by high energy consumption and cost compared to LEDs [74].

## 4. Materials and Methods 

### 4.1. Bacterial Strains and Pre-Culture Preparation

A single colony of the Gram-negative *Pseudomonas fluorescens* (ATCC^®^ 13,525 culti-loops, Thermo Fisher Scientific, Waltham, MA, USA) or Gram-positive *Staphylococcus epidermidis* (NCTC 11,047 Lenticule discs, St. Luis, MI, USA) was placed in 20 mL broth. Tryptic Soy Broth (TSB, VWR Chemicals, Radnor, PA, USA) and Luria Bertani (LB, Becton Dickinson, Franklin Lakes, NJ, USA) supplemented with 5 g L^−1^ NaCl, were used for *P. fluorescens* and *S. epidermidis*, respectively. The pre-cultures were grown at the constant temperature of 25 °C (*P. fluorescens*) and 37 °C (*S. epidermidis*) for 24 h, with shaking (160 rpm).

### 4.2. Biofilm Growth

Bacterial biofilms were grown on the polystyrene surface of petri dishes (50 mm diameter, 8 mm height, Simport, Canada). 400 µL of inoculum suspension, of approximately 10^7^ CFU mL^−1^, was spread on a circular area (7.0 cm^2^) with a plastic loop. After inoculation, the petri dishes were closed and left to grow for 24 h at a constant temperature of 25 °C (*P. fluorescens*) and 20 °C (*S. epidermidis*), under sterile conditions.

### 4.3. Rinsing Procedure

After 24 h, the mature biofilms had grown at the solid–liquid interface. The excess of bacterial suspension was removed using a pipette. 3 mL of Phosphate Buffer Saline (PBS, Sigma Aldrich, Saint Louis, MO, USA) solution was poured into the petri dish and then gently shaken. Finally, the PBS was carefully removed. The rinsing procedure was repeated twice. The biofilm was then ready to be treated using light or for quantification.

### 4.4. Light Set up and Optical Conditions

**Figure 5:**
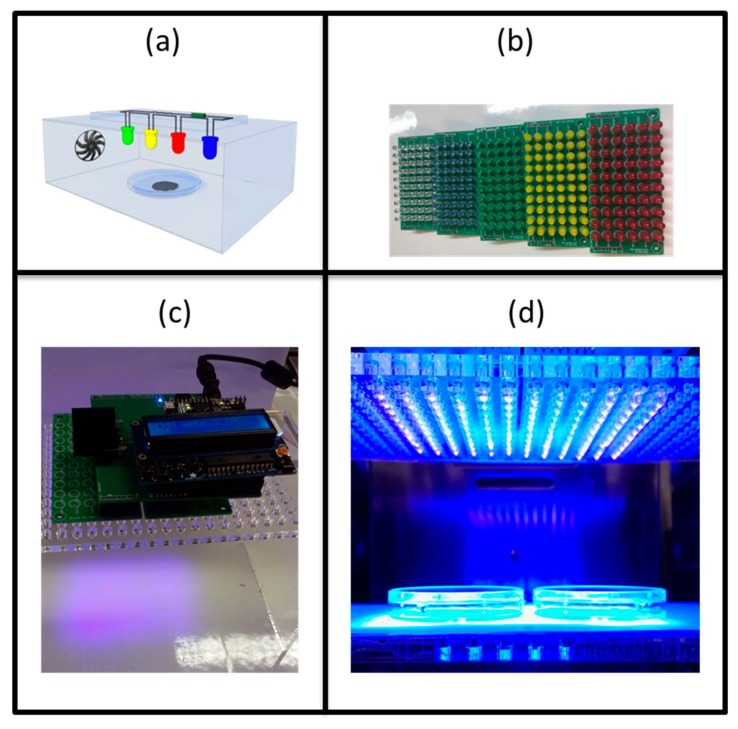
Optical set up: (**a**) scheme of the set up inside the incubator, (**b**) mountable matrices used in the experiments, from right to left the light colors are violet, blue, green, yellow and red, (**c**) set up with liquid-crystal display on the top and (**d**) cross-section of the set up in which the LED matrices are irradiating the biofilm in the petri dishes.

An array composed of 120 LEDs was used as the light source for the investigation. The violet LEDs (UVC5TZ-400-30) were purchased from Bivar (California) while the blue (L-53MBD), green (L-1503CGDK), yellow (L-53YD) and red (L-53HD) were purchased from Kingbright (Taiwan). All of them had an emission in the visible region of the light spectrum. They were optically characterized in terms of wavelength, irradiance and emission in time. The setup was provided with a mechanism that allowed for the replacement of the LED array. A liquid crystal display allowed the operator to change the electric current flowing through the LEDs (0–30 mA), and thus, the power density emitted by the light source. Different conditions were investigated for each color: 100%, 75% and 25% of the maximum irradiances (I_max_) were tested on the bacterial biofilms. For the violet LED array, 2.5% was explored as well, to obtain an irradiance comparable to the other LED sources. The violet-emitted irradiance was measured using a radiometer RM-12 and sensor (UVA+ 339–440 nm) from Opsytec Dr. Grobel (Germany). The spectrometer MF250N from UPRtek Corp. (Taiwan) was used for the other LED colors. The total dose for each condition was calculated as follows [51]:

Total dose = Irradiance × t_TOT_ (1)

where t_TOT_ represents the total exposure time equal to 4 h. It represents the total energy delivered for the irradiated area. Figure 5 displays a scheme of the set up (Figure 5a), two pictures of the set up (top, Figure 5c, and gross section, Figure 5d) and the LEDs matrices (Figure 5b) used during the experiment.

### 4.5. Biofilm Treatments

Mature biofilms were treated for different light exposure times: 30, 60, 90, 120, 150, 210 and 240 min. The distance array-samples equaled 6.5 cm and the temperature during the whole experiment was kept constant at 20 °C by placing the set up inside an incubator. The biofilms were placed open underneath the LED array. The control referred to biofilms in the dark that did not undergo the light treatment. The control biofilms were placed (open) inside the incubator and could be dried by the air.

### 4.6. Biofilm Quantification

After the treatment, 2 mL of PBS solution was added to each biofilm. The biofilm was detached from the surface using a cell scraper (20 mm blade, Carl Roth, Karlsruhe, Germany). Afterwards, the suspension was homogenized with a pipette, it became overall turbid and no pellicles were floating in it. Serial dilution suspensions were prepared in saline solution (0.85% NaCl) and these suspensions were plated both on general medium (GM) and on selective medium (SM). Tryptic Soy Agar (TSA) and CFC-supplemented-Pseudomonas Agar Base (Cetrimide-Fucidin-Cephalosporin-PAB, Thermo Scientific, Waltham, Massachusetts, United States) were used as GM and SM for *P. fluorescens*. While for *S. epidermidis*, brain heart infusion (BHI) supplemented with 13 g L^−1^ of bacteriological agar (Merck, Darmstadt, Germany) and Staphylococcus agar containing 75 g L^−1^ NaCl were used as GM and SM, respectively.

### 4.7. Inactivation Model

The Geeraerd et al. inactivation model was used to fit the light-response data [62]. This non-linear model is based on a sigmoidal-like curve with a shoulder (lag phase), a log-linear inactivation and a tail (indicating the existence of a residual population surviving the inactivation treatment). The data collected in this work did not show any evidence of a shoulder, so the model was simplified to the model without shoulder expressed by the following (static) equation:

(2) $$N\left(t\right)=\left(N_{0}-N_{res}\right)\times exp\left(-k_{max}\times t\right)+N_{res}$$

or in some cases, to the model without shoulder and without tail, which was reduced to the classical log-linear regression model:

(3) $$N\left(t\right)=N_{0}\times exp\left(-k_{max}\times t\right).$$

In these equations, *N(t)* (CFU cm^−2^) represents the biofilm microbial population at time *t* (min), *N*_0_ (CFU cm^−2^) represents the initial cell density, *k_max_* (min^−1^) is the maximum inactivation rate and *N_res_* (CFU cm^−2^) is the biofilm residual population. The difference between the initial population (*N*_0_) and the residual population (*N_res_*) is referred to as log_10_ reduction. During parameters estimation, logarithmically transformed data are used. The root mean squared error (RMSE) between the observed (*N_exp_*) and predicted (*N_mod_*) log_10_ values was used to evaluate the goodness of the fit,

(4) $$\mathrm{RMSE}=\sqrt{\frac{\sum_{1}^{n}{\left(N_{exp}\left(ti\right)-N_{mod}\left(ti\right)\right)}^{2}}{n-p}},$$

where *n* is the number of measurements and *p* is the number of estimated parameters.

### 4.8. Sublethal Injury

The sublethal injury (SI), consequent to light exposure, was calculated using the following equation [75]:

(5) $$\mathrm{Sublethal}\;\mathrm{Injury}\left(\%\right)=\frac{\mathrm{Cell}\;\mathrm{Counts}\;\mathrm{on}\;\mathrm{GM}\;-\;\mathrm{Cell}\;\mathrm{Counts}\;\mathrm{on}\;\mathrm{SM}\;}{\mathrm{Cell}\;\mathrm{Counts}\;\mathrm{on}\;\mathrm{GM}}\times 100\%.$$

SI is calculated based on the continuous fit of the Geeraerd et al. model [62]. It gives a percentage related to the damage caused by light exposure.

### 4.9. Dose for 1 log_10_ Reduction

The estimation of the parameter *N_res_* of the control (no light exposure) and of the light-exposed biofilms allowed for the calculation of the light dose (J cm^−2^) required to reduce the bacterial population with 1 log_10_ (90%). The required dose gave an idea of the strength of the killing potential of the light treatment. The dose for 1 log_10_ reduction has been calculated as:

(6) $$\mathrm{Dose}\;\mathrm{for}\;1\log_{10}\mathrm{red}\;=\frac{\mathrm{Total}\;\mathrm{dose}}{N_{res}\;\mathrm{control}-N_{res}\;\mathrm{light}}$$

where *N_res_* was estimated by the fitting of the Geeraerd et al. model [62].

### 4.10. Statistical Analysis

To statistically compare the estimated parameters evaluated using the Geeraerd et al. model, a one-way analysis of variance (ANOVA) was carried out using a multiple comparison tests based on the *multcompare* function implemented in MATLAB R2016a (The Math Works, Inc.) [62]. A confidence level of 95% was used to assess the significant difference. A *p*-value less than 0.05 was considered significantly different.

## 5. Conclusions

The number of photodynamic inactivation studies has greatly increased in recent years. These studies have mainly examined planktonic cells [38,39,40,41,43,45,46,47,48,49,50,51,52], while few studies have taken bacterial biofilms into consideration [16,30,32,50,51,52,53,54,55]. Since biofilms pose a major global healthcare problem, the impact of photodynamic inactivation on them is both crucial and innovative.

Previous research on the antimicrobial potential of visible light has focused mainly on bacteria growing in a planktonic form and to a lesser extent, on biofilms, which have been identified as a crucial problem for hygiene control in households, industries and clinical environments in the last years. Therefore, this research focused on the effect of five different wavelengths of visible LED light on the inactivation dynamics of mature biofilms. Moreover, this research included both a Gram-negative and Gram-positive bacterial strain, as the Gram-nature is seen as an important factor in the resistance against antimicrobial treatments. This work adds to a growing body of research on the effect of photodynamic inactivation against biofilms. The broad study explores the effect of several wavelengths and irradiance levels on common biofilm-formers with a different Gram-nature. As such, it provides insights into (the limits of) the potential of LED light as an antibiofilm strategy.

The results demonstrated that the high-energy violet light was able to inactivate biofilms of both the Gram-negative *P. fluorescens* and Gram-positive *S. epidermidis*. Blue light also had an inactivating effect on *P. fluorescens* but not on *S. epidermidis*. This demonstrates the higher resistance of the Gram-positive biofilms. Green, yellow and red light had no effect on the biofilm population compared to untreated control samples. Even though biofilms are more antimicrobial-resistant than planktonic bacteria, this research demonstrated the potential of simple visible light as an anti-biofilm strategy. However, the research also demonstrated that this potential is limited to violet light when considering a treatment that should be effective against a broad spectrum of bacteria.

Bacterial inactivation by photons is hypothesized to be mediated by reactive oxygen species. Future work should focus on improving the understanding of the inactivation mechanism of visible light. Such an improved understanding will allow further finetuning of this promising technology.

## Figures and Tables

**Table 1:** Light and treatments’ characterization. Peak wavelengths (λ_max_), values of full width at half maximum (Δλ), irradiances, total doses (related to the different Light-Emitting Diodes (LED) arrays: violet, blue, green, yellow and red) and treatments.

Percentage Maximum Irradiance (I_max_)	Irradiance (mW cm^−2^)	Total Light Dose (J cm^−2^)
	Violet (λ_max_/Δλ 400/20 nm)	
100%	29.2	420.5
75%	21	302.4
25%	6.9	99.4
2.5%	0.7	10.1
	Blue (λ_max_/Δλ 420/60 nm)	
100%	0.49	6.9
75%	0.42	6
25%	0.17	2.5
	Green (λ_max_/Δλ 570/20 nm)	
100%	0.26	3.7
75%	0.22	3.2
25%	0.11	1.6
	Yellow (λ_max_/Δλ 584/35 nm)	
100%	0.14	2
75%	0.11	1.6
25%	0.043	0.6
	Red (λ_max_/Δλ 698/95 nm)	
100%	0.35	5
75%	0.28	4
25%	0.11	1.7

**Table 2:** *P. fluorescens* parameters. Estimated parameters with standard errors of *P. fluorescens* inactivation using the Geeraerd et al. model are listed, i.e., *N*_0_, *N_res_*, *k_max_*, log_10_ reduction and root mean squared error (RMSE) for general and selective media [62].

		General Medium					Selective Medium				
LED	I_max_ (%)	Log_10_ *N*_0_ (Log(CFU cm^−2^))	Log_10_ *N_res_* (Log(CFU cm^−2^))	*k_max_* (min^−1^)	Log_10_ Reduction (Log(CFU cm^−2^))	RMSE	Log_10_ *N*_0_ (Log(CFU cm^−2^))	Log_10_ *N_res_* (Log(CFU cm^−2^))	*k_max_* (min^−1^)	Log_10_ Reduction (Log(CFU cm^−2^))	RMSE
Control	0	^a^ 7.781 ± 0.200	^a^ 3.907 ± 0.182	^a^ 0.072 ± 0.009	^a^ 3.873 ± 0.270	0.4926	^a^ 7.629 ± 0.170	^a^ 4.006 ± 0.142	^a^ 0.077 ± 0.009	^a,e^ 3.623 ± 0.222	0.4123
Violet	2.5	^a^ 7.478 ± 0.208	^b^ 3.275 ± 0.222	^a^ 0.065 ± 0.007	^a^ 4.203 ± 0.304	0.5263	^a^ 7.562 ± 0.424	^a^ 1.200 ± 0.325	^a^ 0.148 ± 0.023	^a^ 3.479 ± 0.236	0.4311
	25	^a^ 7.823 ± 0.215_a_	^c^ 2.517 ± 0.170_a_	^a^ 0.132 ± 0.013_a_	^c^ 5.307 ± 0.274	0.5125	^a^ 7.769 ± 0.272_a_	^c^ 1.227 ± 0.217_a_	^a^ 0.157 ± 0.015_a_	^c^ 5.532 ± 0.307	0.5752
	75	^a^ 7.778 ± 0.311_a_	^c^ 2.158 ± 0.221_a_	^a^ 0.173 ± 0.024_a_	^c^ 5.619 ± 0.382	0.7229	^a^ 7.724 ± 0.245_a_	^b^ 2.192 ± 0.186_a_	^a^ 0.148 ± 0.017_a_	^b^ 6.543 ± 0.348	0.6525
	100	^a^ 7.804 ± 0.394_a_	^d^ 1.000 ± 0.329_a_	^a^ 0.134 ± 0.018_a_	^b^ 6.804 ± 0.513	0.9002	^a^ 7.390 ± 0.176_a_	^b^ 3.911 ± 0.157_a_	^a^ 0.067 ± 0.008_a_	^b^ 6.362 ± 0.534	0.9407
Blue	25	^a^ 7.828 ± 0.182_a_	^b^ 4.583 ± 0.195_b_	^a^ 0.053 ± 0.007_a_	^a,c^ 3.245 ± 0.266	0.4926	^a^ 7.467 ± 0.264^a^	^c^ 2.144 ± 0.391_b_	^a^ 0.065 ± 0.007_a_	^a^ 2.977 ± 0.275	0.4879
	75	^a^ 7.838 ± 0.232_a_	^b^ 4.794 ± 0.150_b_	^a^ 0.126 ± 0.028_a_	^c^ 3.044 ± 0.277	0.5279	^a^ 7.545 ± 0.225_a_	^a,c^ 4.167 ± 0.148_b,c_	^a^ 0.130 ± 0.024_a_	^a^ 3.378 ± 0.269	0.5142
	100	^a^ 7.701 ± 0.237_a_	^c^ 2.327 ± 0.327_b_	^a^ 0.068 ± 0.007_a_	^b^ 5.374 ± 0.404	0.6217	^a^ 7.567 ± 0.198_a_	^b^ 4.591 ± 0.191_b_	^a^ 0.054 ± 0.009_a_	^b^ 5.323 ± 0.472	0.6978
Green	25	^a^ 7.808 ± 0.109_a_	^b^ 4.693 ± 0.078_b_	^a^ 0.090 ± 0.009_a_	^b,c^ 3.115 ± 0.134	0.2531	^a^ 7.554 ± 0.155_a_	^a,b^ 4.304 ± 0.120_b_	^a^ 0.080 ± 0.010_a_	^a,b^ 3.219 ± 0.181	0.3462
	75	^a^ 7.820 ± 0.207_a_	^b^ 4.920 ± 0.136_c_	^a^ 0.113 ± 0.023_a_	^c^ 2.900 ± 0.248	0.4726	^a^ 7.635 ± 0.218_a_	^b^ 4.802 ± 0.147_c_	^a^ 0.103 ± 0.022_a_	^b^ 2.833 ± 0.263	0.5016
	100	^a^ 7.724 ± 0.158_a_	^a^ 4.063 ± 0.123_c_	^a^ 0.087 ± 0.009_a_	^a,b^ 3.660 ± 0.200	0.3775	^a^ 7.618 ± 0.151_a_	^a,b^ 4.399 ± 0.101_c_	^a^ 0.118 ± 0.015_a_	^a,b^ 3.251 ± 0.196	0.3680
Yellow	25	^a^ 7.841 ± 0.223_a_	^b^ 4.954 ± 0.210_b_	^a^ 0.054 ± 0.010_a_	^b^ 2.888 ± 0.306	0.5457	^a^ 7.604 ± 0.105_a_	^b^ 4.197 ± 0.081_b_	^a^ 0.084 ± 0.006_a_	^b^ 2.828 ± 0.342	0.6230
	75	^a^ 7.950 ± 0.268_a_	^b^ 4.828 ± 0.215_c_	^a^ 0.072 ± 0.015_a_	^b^ 3.122 ± 0.344	0.6421	^a^ 7.725 ± 0.267_a_	^a,b^ 4.508 ± 0.245_c_	^a^ 0.066 ± 0.014_a_	^a,b^ 3.217 ± 0.362	0.6478
	100	^a^ 7.818 ± 0.104_a_	^a^ 4.033 ± 0.082_c_	^a^ 0.097 ± 0.007_a_	^a^ 3.785 ± 0.132	0.2453	^a^ 7.582 ± 0.257_a_	^a^ 4.754 ± 0.226_c_	^a^ 0.058 ± 0.013_a_	^a,b^ 3.407 ± 0.132	0.2494

^a,b,c,d^ Effect of the color: For each percentage of I_max_, estimated parameters bearing different subscripts following the values (no letter in common) are significantly different (*p* ≤ 0.05). Effect of the percentage of I_max_: For each color, estimated parameters bearing different superscripts in front of the values (no letter in common) are significantly different (*p* ≤ 0.05).

**Table 3:** *S. epidermidis* parameters. Estimated parameters with standard errors of *S. epidermidis* inactivation using the Geeraerd et al. model are listed, i.e., *N*_0_, *N_res_*, *k_max_*, log_10_ reduction and RMSE for general and selective media [62].

		**Geeraerd et al. Model** [62]									
		**General Medium**					**Selective Medium**				
**LED**	**I_max_ (%)**	**Log_10_*N*_0_ (Log(CFU cm^−2^))**	**Log_10_*N_res_* (Log(CFU cm^−2^))**	***k_max_* (min^−1^)**	**Log_10_ reduction (Log(CFU cm^−2^))**	**RMSE**	**Log_10_*N*_0_ (Log(CFU cm^−2^))**	**Log_10_*N_res_* (Log(CFU cm^−2^))**	***k_max_* (min^−1^)**	**Log_10_ reduction (Log(CFU cm^−2^))**	**RMSE**
Control	0	^a^ 6.844 ± 0.208	^a^ 6.275 ± 0.125	^a^ 0.125 ± 0.446	^a^ 0.569 ± 0.242	0.4645	^a^ 6.900 ± 0.244	^a^ 6.132 ± 0.162	^a^ 0.056 ± 0.060	^a^ 0.768 ± 0.293	0.5486
Violet	2.5	^a^ 6.847 ± 0.180	^a^ 6.254 ± 0.112	^a^ 0.076 ± 0.100	^a^ 0.593 ± 0.212	0.3408	^a^ 6.771 ± 0.135	^b^ 3.762 ± 0.157	^a^ 0.046 ± 0.005	^b,c^ 3.008 ± 0.207	0.4023
	25	^a^ 6.859 ± 0.219_a_	^b^ 5.687 ± 0.179	^a^ 0.038 ± 0.021_a_	^a^ 1.172 ± 0.283	0.5097	^a^ 6.842 ± 0.209_a_	^c^ 4.382 ± 0.219	^a^ 0.043 ± 0.009_a_	^b^ 2.460 ± 0.303	0.5182
	75	^a^ 6.879 ± 0.193_a_	^c^ 4.729 ± 0.140	^a^ 0.076 ± 0.019_a_	^b^ 2.149 ± 0.238	0.4441	^a^ 6.816 ± 0.164_a_	^b,c^ 4.086 ± 0.143	^a^ 0.066 ± 0.010_a_	^b^ 2.730 ± 0.217	0.3894
	100	^a^ 6.923 ± 0.258_a_	^d^ 3.232 ± 0.189	^a^ 0.111 ± 0.020_a_	^c^ 3.690 ± 0.320	0.6000	^a^ 6.892 ± 0.193_a_	^b^ 3.516 ± 0.145	^a^ 0.098 ± 0.014_a_	^c^ 3.376 ± 0.293	0.4500
		**Geeraerd et al. Model, Reduced to Log-Linear Regression**									
		**General Medium**					**Selective Medium**				
**LED**	**I_max_ (%)**	**Log_10_*N*_0_ (Log(CFU cm^−2^))**		***k_max_* (min^−1^)**		**RMSE**	**Log_10_*N*_0_ (Log(CFU cm^−2^))**		***k_max_* (min^−2^)**		**RMSE**
Control	0	^a^ 6.574 ± 0.174		^a^ 1.5 × 10^−3^ ± 1.3 × 10^−3^		0.5002	^a^ 6.674 ± 0.201		^a^ 2.8 × 10^–3^ ± 1.5 × 10^−3^		0.5786
Blue	25	^a^ 6.564 ± 0.099_a_		^a^ 1.3 × 10^−3^ ± 7.0 × 10^−4^_a_		0.2963	^a^ 6.628 ± 0.104_a_		^a^ 2.2 × 10^−3^ ± 8.0 × 10^−4^_a_		0.3110
	75	^a^ 6.787 ± 0.123_a_		^a^ 4.4 × 10^−3^ ± 9.0 × 10^−4^_a_		0.3534	^a^ 6.830 ± 0.140_a_		^a^ 5.2 × 10^−3^ ± 1.1 × 10^−3^_a_		0.4019
	100	^a^ 6.735 ± 0.058_a_		^a^ 3.1 × 10^−3^ ± 4.0 × 10^−4^_a_		0.2568	^a^ 6.735 ± 0.093_a_		^a^ 3.0 × 10^−3^ ± 7.0 × 10^−4^_a_		0.1671
Green	25	^a^ 6.728 ± 0.159_a_		^a^ 3.5 × 10^−3^ ± 1.2 × 10^−3^_a_		0.4632	^a^ 6.714 ± 0.163_a_		^a^ 3.6 × 10^−3^ ± 1.2 × 10^−3^_a_		0.4586
	75	^a^ 6.815 ± 0.091_a_		^a^ 2.0 × 10^−3^ ± 7.0 × 10^−4^_a_		0.2769	^a^ 6.882 ± 0.096_a_		^a^ 2.1 × 10^−3^ ± 7.0 × 10^−4^_a_		0.2627
	100	^a^ 6.922 ± 0.139_a_		^a^ 3.7 × 10^−3^ ± 1.1 × 10^−3^_a_		0.4645	^a^ 6.970 ± 0.161_a_		^a^ 4.3 × 10^−3^ ± 1.2 × 10^−3^_a_		0.4008
Yellow	25	^a^ 6.499 ± 0.132_a_		^a^ 2.0 × 10^−3^ ± 1.0 × 10^−3^_a_		0.3632	^a^ 6.559 ± 0.126_a_		^a^ 2.5 × 10^−3^ ± 9.0 × 10^−4^_a_		0.3798
	75	^a^ 6.779 ± 0.112_a_		^a^ 2.4 × 10^−3^ ± 8.0 × 10^−4^_a_		0.3374	^a^ 6.763 ± 0.117_a_		^a^ 2.5 × 10^−3^ ± 8.0 × 10^−4^_a_		0.3222
	100	^a^ 6.703 ± 0.122_a_		^a^ 2.3 × 10^−3^ ± 9.0 × 10^−4^_a_		0.3497	^a^ 6.716 ± 0.121_a_		^a^ 2.7 × 10^−3^ ± 9.0 × 10^−4^_a_		0.3505
Red	25	^a^ 6.812 ± 0.120_a_		^a^ 1.6 × 10^−3^ ± 4.0 × 10^−4^_a_		0.3478	^a^ 6.796 ± 0.121_a_		^a^ 1.0 × 10^−3^ ± 9.0 × 10^−4^_a_		0.345
	75	^a^ 6.989 ± 0.195_a_		^a^ 4.1 × 10^−3^ ± 1.5 × 10^−3^_a_		0.6212	^a^ 7.064 ± 0.219_a_		^a^ 4.8 × 10^−3^ ± 1.7 × 10^−3^_a_		0.5626
	100	^a^ 6.772 ± 0.081_a_		^a^ 1.0 × 10^−3^ ± 6.0 × 10^−4^_a_		0.3034	^a^ 6.778 ± 0.105_a_		^a^ 1.1 × 10^−3^ ± 7.0 × 10^−4^_a_		0.2341

^a,b,c,d^ Effect of the color: For each percentage of I_max_, estimated parameters bearing different superscripts following the values (no letter in common) are significantly different (*p* ≤ 0.05). Effect of the percentage of I_max_: For each color, estimated parameters bearing different subscripts in front of the values (no letter in common) are significantly different (*p* ≤ 0.05).
